# An Appreciative Husband

**Published:** 1889-05

**Authors:** 


### AN APPRECIATIVE HUSBAND.

"Talk about wives," said Farmer Hawbuck. "I've got one wife in a million. Why, she gits up in the mornin', milks seventeen cows, and gits breakfast for twenty hard workin' men before six o'clock." "She must be a very robust woman, Hawbuck," remarked one of his hearers. "On the contrairy," put in the farmer, "she is pale and delikit-like. Gosh, ef that woman was strong I dunno what work she couldn't do."

